# Light Chain Amyloidosis: Epidemiology, Staging, and Prognostication

**DOI:** 10.14797/mdcvj.1070

**Published:** 2022-03-14

**Authors:** Kelty R. Baker

**Affiliations:** 1 Houston Methodist Hospital, Houston, Texas, US

**Keywords:** light chain amyloidosis, prognosis, Mayo Clinic staging system, cardiac amyloidosis

## Abstract

Amyloidosis is a disorder of protein misfolding and metabolism in which insoluble fibrils are deposited in various tissues, causing organ dysfunction and eventually death. Out of the 60-plus heterogeneous amyloidogenic proteins that have been identified, approximately 30 are associated with human disease. The unifying feature of these proteins is their tendency to form beta-pleated sheets aligned in an antiparallel fashion. These sheets then form rigid, nonbranching fibrils that resist proteolysis, causing mechanical disruption and local oxidative stress in affected organs such as the heart, liver, kidneys, nervous system, and gastrointestinal tract. Here we review the epidemiology of light chain amyloidosis, the staging, and the concomitant prognostication that is critical in determining the appropriate treatment.

## Introduction

The amyloidoses are disorders of protein misfolding and metabolism in which insoluble fibrils are deposited in various tissues, causing organ dysfunction and eventually death. More than 60 heterogeneous amyloidogenic proteins have been identified, with approximately 30 of these known to be associated with human disease.^1,2^ The unifying feature of these proteins is their tendency to form beta-pleated sheets aligned in an antiparallel fashion. These sheets then form rigid, nonbranching fibrils that resist proteolysis, causing mechanical disruption and local oxidative stress in affected organs such as the heart, liver, kidneys, nervous system, and gastrointestinal tract.^3^ The following pages review the epidemiology of light chain amyloidosis as well as staging and the concomitant prognostication so important in determining the appropriate treatment for this serious systemic illness.

## Epidemiology

Primary or light chain (AL) amyloidosis, the most common type of systemic amyloidosis, occurs when the free light chains normally associated with immunoglobulins are produced in excess by clonal or frankly malignant plasma cells. Although AL amyloidosis is not considered a cancer, it shares some similar characteristics and treatments with multiple myeloma. AL amyloidosis is most commonly diagnosed when the affected patient has less than 10% bone marrow plasma cells, the quantity required to make a diagnosis of myeloma, but may also occur in association with full-blown multiple myeloma, non-Hodgkin’s lymphoma, Waldenström’s macroglobulinemia, chronic lymphocytic leukemia, Sjogren’s syndrome, and Behçet syndrome.^4^ It is a relatively rare condition, with a worldwide incidence of 5.1 to 12.8 cases per million person-years. In the United States, between 1,275 and 3,200 new cases are diagnosed annually,^5^ yielding an incidence of approximately 9 to 14 cases per million person-years and a prevalence of 40.5 cases per million as of 2015—a significant increase from the prevalence of 15.5 cases per million reported in 2007. It is hypothesized that this rising prevalence is due to a growing awareness of the disease and its manifestations as well as improved treatments that have lowered the mortality rate.^6^

The age-specific incidence rate of AL amyloidosis increases with each decade over age 40 years, with 64 years being the median age at diagnosis. In fact, less than 5% of affected patients are under the age of 40. As with multiple myeloma, afflicted males outnumber females 3:2. Surprisingly, although incidence rates of myeloma are over twofold higher in Black patients than White patients, and systemic amyloidosis is a related plasma cell disorder, AL amyloidosis has no apparent ethnic or geographic specificity. Potential reasons for this interesting discrepancy could be due to the fact that the largest population-based studies of AL amyloidosis have consisted of predominantly White patients, and to the inequitable access of Black patients to specialty physicians, appropriate diagnostic testing, and tertiary care center referral. Systematic underdetection is also thought to be a large part of the problem because the structural heart changes, proteinuria, and neuropathy seen with AL amyloidosis can be misattributed to hypertension or diabetes mellitus, both of which are more prevalent in racial minorities.^7^

Once they are evaluated, non-Hispanic Black patients are more likely to present with a dFLC (the difference between involved and uninvolved serum free light chains) > 180 mg/L compared with other ethnic groups (39% vs 22–33%, *P* = .044) and to have a higher prevalence of cardiac involvement (69% of patients), whereas renal involvement is more prevalent in non-Hispanic White patients (78% of patients). On the other hand, Hispanic patients have a significantly higher median B-type natriuretic peptide (BNP) at baseline than other ethnicities (BNP 1,041 vs 221–480 pg/mL, *P* = .001), with the majority having a level > 700 pg/mL and resultant higher numbers of patients with cardiac stage IIIB disease. Whether these ethnic differences are due to delayed disease recognition by physicians, variances in how quickly symptoms are reported by patients, or racial differences in the pathogenesis of amyloidosis is uncertain. For instance, while we know that germline expression of the *IGLV1-44* gene is associated with cardiac tropism and the *IGLV6-57* gene is associated with renal tropism, variances in gene expression with regard to race or ethnicity haven’t been assessed.^7^

## Staging

As with other plasma cell dyscrasias, staging of amyloidosis is performed at diagnosis to help determine a patient’s prognosis and the most appropriate course of treatment. Staging is based on the presence and severity of heart damage, which is assessed by serum levels of cardiac troponin T (TnT) and N-terminal (NT)-pro hormone BNP (NT-proBNP), or more rarely troponin I and BNP. Since the severity of heart damage has been determined to be the most important factor in predicting survival, measurement of these cardiac biomarkers forms the basis of all AL staging systems. Of note, while hematologists tend to use the terms NT-proBNP and BNP interchangeably, they are different molecules. BNP is a biologically active hormone secreted by cardiac myocytes in response to increased blood volume and resultant wall stress, and it is created when a specific convertase cleaves NT-proBNP from the preprohormone. Because NT-proBNP is then cleared passively from the body, it has a longer half-life, circulates at higher concentrations in the bloodstream, and is less influenced by acute hemodynamic variations than BNP.^8^

The prognostic utility of the most popular staging systems has been well validated for both newly diagnosed patients and those in first relapse.^9^ Recently, a study from the Mayo Clinic found it useful to reassess a patient’s stage at 3 and 6 months after initiating treatment because migration to a higher stage predicts a poor prognosis.^10^ Although we have seen significant improvement in patient survival due to a combination of earlier diagnosis, more effective therapies, and lower transplant-related mortality, the most widely used staging schema (reviewed below) continue to predict survival at a variety of timepoints in a given patient’s treatment course.

### European Modification of Mayo 2004 Staging System

The Mayo 2004 staging system uses a TnT cutoff level of 0.035 mcg/L and NT-proBNP level of 332 ng/L to place AL amyloidosis patients into three groups: (1) stage I, normal levels of both, (2) stage II, an elevated level of either but not both, and (3) stage III, elevated levels of both. The resultant median survival for those patients not undergoing hematopoietic cell transplantation (HCT) in a related study was 26, 11, and 4 months for stages I, II, and III, respectively.^11^ The Europeans subsequently proposed splitting stage III into two groups depending on the absence (stage IIIA) or presence (stage IIIB) of an NT-proBNP level > 8500 ng/L.^12^ For laboratories that use troponin I in place of TnT, the cutoff value is ≥ 0.10 mcg/L. For laboratories using a high-sensitivity troponin T assay, a cutoff of 40 pg/mL should be used.

### Mayo 2012 Staging System

In 2012, the Mayo Clinic updated their staging system to include the dFLC (with > 18 mg/dL being significant), and changing the cardiac biomarkers to an NT-proBNP > 1800 ng/L and a cardiac TnT > 0.025 mcg/L. Stage I encompasses those patients with no elevated risk factors, while stages II, III, and IV encompass those who have one, two, or three elevated risk factors, respectively. The resultant median overall survival from the time of diagnosis for these patients was 94, 40, 14, and 6 months. For patients fit enough to undergo HCT, the 4-year estimated survival rates were not reached for those with stage I disease and were 97, 58, and 22 months for those with stages II, III, and IV disease, respectively.^13^

## Prognosis

When detected at a late stage, AL amyloidosis has a poor long-term prognosis. Median survival can be as short as 5 months, with infection and cardiac or hepatic failure being the most common causes of death.^14^ Important factors in determining prognosis are summarized below and divided into those that are assessed before treatment initiation and those reassessed following the completion of therapy.

## Pretreatment Variables

### Laboratory Parameters

The ability to detect circulating FLCs dramatically changed the landscape of AL amyloidosis treatment, allowing for the earlier detection of disease and improved prognostication of patient outcomes. In addition to pioneering the use of the involved FLC (iFLC) level and FLC ratio, the Mayo group also advocated for analysis of the dFLC. Higher levels of dFLC at the time of diagnosis are associated with an increased plasma cell burden, greater severity of gastrointestinal disease, and greater likelihood of renal insufficiency and cardiac involvement as well as greater severity of said heart disease as manifested by lower ejection fractions and higher levels of NT-proBNP and TnT. Patients with a higher dFLC (> 29.4 mg/dL for kappa disease and > 18.2 mg/dL for lambda disease) had a significantly worse overall survival (OS) of 10.9 months as opposed to 37.1 months for those with a lower dFLC (*P* < .001). Similar results were seen when patients with normal and abnormal FLC ratios were compared: the former group had a median OS of 65.6 months while the latter group’s median OS was only 16.2 months (*P* < .001). Finally, they reported that those without an identifiable heavy chain were more likely to have increased levels of dFLC and posited that unbound light chains may be inherently more prone to the misfolding characteristic of AL amyloidosis.^15^

Immunoparesis, the term used to describe the low levels of uninvolved immunoglobulins seen in 66% of AL amyloid patients at the time of diagnosis, is associated with a diminished responsiveness to first-line therapy,^16^ a shorter progression-free survival (PFS) but not OS,^17,18^ and decreased responsiveness to HCT that can be overcome by the use of pretransplantation immunomodulatory drugs and bortezomib.^19^

Not unexpectedly, those with lower levels of dFLC at diagnosis (< 50 mg/dL) have a lower burden of disease as manifested by a smaller monoclonal protein spike, fewer bone marrow plasma cells, a better Karnofsky performance status, greater fitness for HCT,^20^ lower frequency and severity of cardiac involvement, and a smaller number of affected target organs. Interestingly, although these same patients have an increased chance of renal involvement and proteinuria,^20,21^ they respond well to therapy, having an increased chance for a complete response (CR) compared to those with a higher dFLC as well as a lower risk for early death over the same timeframe. In fact, CR was achieved in 57% of patients as opposed to the approximately 40% traditionally seen with currently available therapy, and those in CR had a lower likelihood of needing hemodialysis over the subsequent 3 years.^20^ All of these factors result in a superior progression-free survival and overall survival compared to those patients who had an initial dFLC > 50 mg/dL.^22,23^

Elevated levels of von Willebrand antigen (vWF-Ag) is a newly recognized risk factor for early mortality that requires further validation. Kastritis et al. reported that 76% of patients with AL amyloidosis have a vWF-Ag level above the upper limit of normal, perhaps reflecting endothelial dysfunction arising from the vascular deposition of amyloid fibrils. Those with a vWF-Ag level > 230 U/dL incurred a 3-month mortality of 26% and a 6-month mortality of 45% independent of their NT-proBNP level as opposed to 10% and 17% mortality, respectively, of those not so affected. The significance of this laboratory finding is even more striking in those with more advanced cardiac disease. Patients with stage III cardiac disease who have a vWF-Ag level > 230 U/dL have a 12-month survival of only 17% compared to 68% of those with vWF-Ag levels < 230 U/dL. Even worse, individuals with stage IIIB disease and an elevated vWF-Ag level lived only 2 months compared to 6 months for those with lower levels.^24^

An elevated D-dimer level > 0.5 mcg/mL can be seen in approximately 50% of AL amyloidosis patients and has recently been reported to adversely impact survival. Patients with a D-dimer level < 0.5 mcg/mL have a median OS of 5.86 years, those with a level > 0.5 mcg/mL but < 1 mcg/mL have a median OS of 4.04 years, and those with > 1 mcg/mL have a median survival of only 2.08 years (*P* < .001).^25^ Uric acid levels over 8 mg/dL and an elevated red blood cell distribution width—both easily obtained tests—are also thought to be strong predictors of early death independent of the presence of cardiac disease, although this needs further study and validation.^26,27^

Finally, growth differentiation factor-15 has been reported to increase the risk of early death and results in a poor OS independent of a patient’s levels of NT-proBNP and high-sensitivity TnT. Growth differentiation factor-15 is a member of the transforming growth factor beta family. Also known as macrophage inhibitory cytokine-1, GDF-15 is secreted by cardiac myocytes in response to oxidative stress, ischemia, and mechanical stretch and has been found in other populations to predict cardiovascular mortality and progressive renal disease.^28^

### Pathologic Parameters

Numerous authors have reported on the inverse association historically seen between the degree of bone marrow plasma cell infiltration and survival, with the critical level being > 10%.^29,30,31^ Such patients are more likely to have cardiac involvement, a trend toward higher early mortality, and significantly shorter PFS (median 18 vs 48 months, *P* = .02) and OS (median of 33 months vs not reached). Thankfully, modern therapies appear to overcome this negative prognostic feature.^31^

A recent study from Ohio State University revealed several interesting findings regarding cytogenetic risk factors found on bone marrow analysis. First, deletion or monosomy of 13q detected by fluorescence in situ hybridization (FISH) is associated with the presence of cardiac amyloidosis; surprisingly, however, its presence or absence doesn’t impact survival in affected patients. In contrast, there are no known cytogenetic abnormalities detected by FISH that predict renal involvement at diagnosis. Second, median PFS is impacted by the presence of cytogenetic abnormalities detectable by FISH, where PFS is 6.5 years in those with normal results and only 2.0 years in those with abnormal results. Median OS is affected similarly, being 11.0 years in those with normal FISH results and dropping to 4.3 years in those with abnormal results. This is despite there being no difference in achievement of a CR or very good partial response in these patients. Third, hyperdiploidy (defined in this study both classically with multiple copies of whole chromosomes and also as gains of 2 or more of the following: 5p/5q, 1q21, and 11q23) is associated with lower PFS and OS. Finally, patients with gain of 1q21 who received daratumumab enjoyed an intriguing trend toward improved hematologic response.^32^

Although the 11;14 translocation seen in half of AL amyloidosis patients is historically associated with a statistically significant reduction in PFS,^32,33^ especially if the affected patients undergo bortezomib induction therapy,^34^ this adverse cytogenetic risk appears to be overcome by the use of high-dose melphalan. In fact, patients harboring this translocation have an improved chance of attaining complete remission following autologous stem cell transplantation (CR in 41.2% vs 20% in others without this translocation, *P =* .02) as well as a resultant hematologic event-free survival of 46.1 vs 28.1 months (*P* = .05).^35^ Interestingly, this mutation has a particular predilection for patients with isolated amyloidosis, with a prevalence of 56.5% versus 17.6% in those with underlying multiple myeloma (*P* = .022).^33^ This probably speaks to the differences in plasma cell biology between AL amyloidosis and multiple myeloma and warrants more detailed study.

### Cardiovascular Parameters

A large number of cardiovascular parameters have been investigated for prognostic relevance with regards to AL amyloidosis and are summarized in ***Table 1***.^36,37,38,39,40,41,42,43,44,45,46,47,48^ For those thought to have isolated renal amyloidosis, a PR interval over 160 msec and a QTc interval over 417 msec were both independently associated with all-cause mortality (*P* values of .005 and .004, respectively)^49^ and could therefore serve as a simple way to screen for patients who warrant a more intensive look at their cardiac function prior to initiation of therapy.

**Table 1:** Cardiovascular risk factors for increased light chain amyloidosis mortality.^36,37,38,39,40,41,42,43,44,45,46,47,48^ OS: overall survival; MRI: magnetic resonance imaging; EF: ejection fraction


FINDING	RISK FACTOR	NOTES

**Arrhythmias**	Atrial fibrillation	Inferior short-term OS but no impact on peritransplant mortality^36,37^

	Nonsustained ventricular tachycardia	Inferior short-term OS but no impact on peritransplant mortality^36^

	Ventricular tachycardia	Increased mortality^37^

**Cardiac MRI Findings**	Left atrial EF < 16%	Higher risk of 2-year mortality^41^

	Long axis strain > –7%	Higher risk of death and cardiac transplantation^42^

	Myocardial contraction fraction < 52.6%	Higher risk of death and cardiac transplantation^42^

	Right ventricular dysfunction	Predicts all-cause mortality^43^

	New York Heart Association functional class 3/4	Increased mortality^44^

**Clinical Findings**	Left heart valve thickening > 3 mm	Higher all-cause mortality^45^

**Echocardiographic Findings**	Interventricular septal thickness > 15 mm	Inferior OS^46^

	Relative wall thickness > 0.74	Increased mortality^44^

	Right atrial volume index > 35 and other signs of right ventricular dysfunction	High 1-year mortality^37^

	Global longitudinal strain (GLS) < 17%	5-year survival of 47% vs 95% if GLS > 17%^38,39,40^

**Electrocardiographic Findings**	Abnormal QRS axis	Poorer survival^47^

	Frontal QRS-T angle > 102	Increased mortality^48^

	QTc > 483 msec	Poorer survival^47^


Another cardiovascular parameter potentially useful for predicting prognosis but requiring additional study is measurement of flow-mediated dilatation of the brachial artery using specialized, highly operator-dependent ultrasound technology.^50^

### Clinical Findings

Patients presenting with pleural effusions are more likely to have impaired right ventricular function, increased amyloid burden, and worse outcomes than those with isolated pericardial effusions and those without pleural effusions.^51^

## Post-Treatment Variables

### Laboratory Parameters

In a 2021 paper from the Mayo Clinic group reporting on the outcomes of 1,357 patients with AL amyloidosis, the median OS was 4.0 years (95% CI, 3.3-4.6 years); however, as detailed in ***Table 2***, survival varied significantly based on the stage at diagnosis (*P* < .001 regardless of the staging system used).^10^ Although the majority of patients retained their original staging when reassessed at 3 months, some patients improved by one or more stages while others worsened. The OS at this timepoint was 9.8 to 10.5 years for those who maintained their original stage, 10.5 to 10.8 years for those who improved their stage, and 4.0 years for those who worsened. At 6 months following treatment initiation, approximately half of patients retained their original stage. Overall survival at this timepoint was over 8.1 years in those whose stage had remained stable or improved and 3.8 to 5.1 years in those whose stage had worsened.^10^

**Table 2:** Overall survival (OS) of light chain amyloidosis patients by stage at diagnosis and time from treatment initiation. Group 1: 2015 European modification of Mayo 2004 staging; Group 2: Mayo 2012 staging; NR: not reached; N/A: not assessed


GROUP 1 STAGE (1,357 PTS)	MEDIAN OS AT DIAGNOSIS (YRS)	MEDIAN OS AT 3 MO (YRS)	MEDIAN OS AT 6 MO (YRS)

I	12	11.8	NR

II	5.4	10.8	NR

IIIA	1.8	4.6	5.4

IIIB	0.4	1.1	0.9

GROUP 2 STAGE (1,339 PTS)			

I	11.4	11.8	NR

II	8.2	9.0	NR

III	2.4	5.2	4.6

IV	0.5	0.8	0.9


The significance of an elevated dFLC level and the presence of immunoparesis at diagnosis were previously discussed. Not surprisingly, attainment of a dFLC less than 10 mg/L after treatment (known as astringent dFLC response) is associated with superior survival, increased cardiac and renal responses, and longer time to next treatment.^52^ Similarly, absence of immunoparesis at 1 year after initiation of treatment is an independent marker for long-term survival.^17^

### Pathologic Parameters

The depth of hematologic response to prior therapy is important in determining the likelihood of an end-organ response and prolonged PFS as well as OS in some studies.^53^ Patients with no residual monotypic plasma cells in the bone marrow aspirate as assessed by multiparametric flow cytometry at the end of therapy had a 3-year PFS of 88%, much better than the 28% seen in those with evidence of minimal residual disease (MRD) (*P* < .001). This PFS advantage was especially pronounced in those who achieved both MRD negativity and a hematologic CR (attainment of normal serum protein electrophoresis, urine protein electrophoresis, and serum free light chain ratio). In this fortunate group, 3-year PFS was 100% as opposed to 33% in those who achieved hematologic CR but had residual clonal plasma cells in the bone marrow.^54^

Minimal residual disease negativity also increases the likelihood of end-organ response. Although the number of patients in a recent Mayo Clinic report is small, all patients with cardiac disease that became MRD-negative achieved a cardiac response as opposed to only 83% of those with residual clonal plasma cells at the end of therapy. Similar findings were seen in patients with renal and hepatic disease.^12,54^ Although post-therapy bone marrow aspiration and biopsy is not routinely recommended as part of our current consensus criteria for response assessment, the prognostic benefit appears obvious, and it is likely that future revisions of consensus recommendations will incorporate this relatively new technology.

## Conclusion

AL amyloidosis is a serious multisystem disorder that can quickly progress and become fatal if not detected in a timely fashion. Those with a higher burden of disease as manifested by high levels of free light chains, increased numbers of bone marrow plasma cells, and more severe end-organ involvement are more likely to experience treatment failure and diminished survival. It is important that careful staging and assessment of prognostic factors be carried out before diagnosis and following the initiation of therapy so that patients can receive the most appropriate treatment and guidance regarding goals of care.

## Key Points

- Primary or light chain (AL) amyloidosis is the most common type of systemic amyloidosis. It is a protein misfolding and metabolism disorder in which insoluble fibrils are deposited in various tissues, causing organ dysfunction and eventually death; therefore, early detection is crucial.
- AL amyloidosis is a relatively rare condition, with only 1,275 to 3,200 new cases diagnosed annually in the United States.
- Patients who have high levels of free light chains and bone marrow plasma cells and severe end-organ involvement are more likely to experience treatment failure and diminished survival.
- Staging of amyloidosis is best performed at diagnosis to help determine a patient’s prognosis and most suitable course of treatment, and then repeated 3 and 6 months after initiating treatment to determine efficacy and response to therapy.

## References

[1] Sipe JD, Benson MD, Buxbaum JN, et al. Amyloid fibril protein nomenclature: 2010 recommendations from the nomenclature committee of the International Society of Amyloidosis. Amyloid. 2010 Sep;17(3-4):101-4. doi: 10.3109/13506129.2010.526812 21039326

[2] Benson MD, Buxbaum JN, Eisenberg DS, et al. Amyloid nomenclature 2020: update and recommendations by the International Society of Amyloidosis (ISA) nomenclature committee. Amyhoid. 2020 Dec;27(4):217-222. doi: 10.1080/13506129.2020.1835263 33100054

[3] Merlini G, Bellotti V. Molecular mechanisms of amyloidosis. N Engl J Med. 2003 Aug 7;349(6):583-96. doi: 10.1056/NEJMra023144 12904524

[4] Comenzo RL. How I treat amyloidosis. Blood. 2009 Oct 8;114(15):3147-57. doi: 10.1182/blood-2009-04-202879 19617578

[5] Falk RH, Comenzo RL, Skinner M. The systemic amyloidoses. N Engl J Med. 1997 Sep 25;337(13):898-909. doi: 10.1056/NEJM199709253371306 9302305

[6] Quock TP, Yan T, Chang E, Guthrie S, Broder MS. Epidemiology of AL amyloidosis: a real-world study using US claims data. Blood Adv. 2018 May 22;2(10):1046-1053. doi: 10.1182/bloodadvances.2018016402 29748430 PMC5965052

[7] Staron A, Connors LH, Zheng L, Doros G, Sanchorawala V. Race/ethnicity in systemic AL amyloidosis: perspectives on disease and outcome disparities. Blood Cancer J. 2020 Nov 10;10(11):118. doi: 10.1038/s41408-020-00385-0 33173025 PMC7655813

[8] Bettencourt P, Azevedo A, Pimenta J, Friões F, Ferreira S, Ferreira A. N-terminal-pro-brain natriuretic peptide predicts outcome after hospital discharge in heart failure patients. Circulation. 2004 Oct 12;110(15):2168-74. doi: 10.1161/01.CIR.0000144310.04433.BE 15451800

[9] Hwa YL, Gertz MA, Kumar SK, et al. Prognostic restaging at the time of second-line therapy in patients with AL amyloidosis. Leukemia. 2019 May;33(5):1268-1272. doi: 10.1038/s41375-019-0400-5 30737485

[10] Abdallah N, Dispenzieri A, Muchtar E, et al. Prognostic restaging after treatment initiation in patients with AL amyloidosis. Blood Adv. 2021 Feb 23;5(4):1029-1036. doi: 10.1182/bloodadvances.2020003782 33595624 PMC7903228

[11] Dispenzieri A, Gertz MA, Kyle RA, et al. Prognostication of survival using cardiac troponins and N-terminal pro-brain natriuretic peptide in patients with primary systemic amyloidosis undergoing peripheral blood stem cell transplantation. Blood. 2004 Sep 15;104(6):1881-7. doi: 10.1182/blood-2004-01-0390 15044258

[12] Palladini G, Paiva B, Wechalekar A, et al. Minimal residual disease negativity by next-generation flow cytometry is associated with improved organ response in AL amyloidosis. Blood Cancer J. 2021 Feb 16;11(2):34. doi: 10.1038/s41408-021-00428-0 33594045 PMC7887224

[13] Kumar S, Dispenzieri A, Lacy MQ, et al. Revised prognostic staging system for light chain amyloidosis incorporating cardiac biomarkers and serum free light chain measurements. J Clin Oncol. 2012 Mar 20;30(9):989-95. doi: 10.1200/JCO.2011.38.5724 22331953 PMC3675680

[14] Kyle RA, Gertz MA, Greipp PR, et al. A trial of three regimens for primary amyloidosis: colchicine alone, melphalan and prednisone, and melphalan, prednisone, and colchicine. N Engl J Med. 1997 Apr 24;336(17):1202-7. doi: 10.1056/NEJM199704243361702 9110907

[15] Kumar S, Dispenzieri A, Katzmann JA, et al. Serum immunoglobulin free light-chain measurement in primary amyloidosis: prognostic value and correlations with clinical features. Blood. 2010 Dec 9;116(24):5126-9. doi: 10.1182/blood-2010-06-290668 20798235 PMC3012533

[16] Muchtar E, Magen H, Itchaki G, et al. Uninvolved immunoglobulins predicting hematological response in newly diagnosed AL amyloidosis. Leuk Res. 2016 Feb;41:56-61. doi: 10.1016/j.leukres.2015.11.013 26658109

[17] Rodríguez-Lobato LG, Fernández de Larrea C, Cibeira MT, et al. Prognostic impact of immunoparesis at diagnosis and after treatment onset in patients with light-chain amyloidosis. Amyloid. 2017 Dec;24(4):245-252. doi: 10.1080/13506129.2017.1390451 29052436

[18] Sachchithanantham S, Berlanga O, Alvi A, et al. Immunoparesis defined by heavy+light chain suppression is a novel marker of long-term outcomes in cardiac AL amyloidosis. Br J Haematol. 2017 Nov;179(4):575-585. doi: 10.1111/bjh.14908 28990174

[19] Muchtar E, Dispenzieri A, Kumar SK, et al. Immunoparesis status in immunoglobulin light chain amyloidosis at diagnosis affects response and survival by regimen type. Haematologica. 2016 Sep;101(9):1102-9. doi: 10.3324/haematol.2016.147041 27479823 PMC5060027

[20] Dittrich T, Bochtler T, Kimmich C, et al. AL amyloidosis patients with low amyloidogenic free light chain levels at first diagnosis have an excellent prognosis. Blood. 2017 Aug 3;130(5):632-642. doi: 10.1182/blood-2017-02-767475 28550043

[21] Milani P, Basset M, Russo F, Foli A, Merlini G, Palladini G. Patients with light-chain amyloidosis and low free light-chain burden have distinct clinical features and outcome. Blood. 2017 Aug 3;130(5):625-631. doi: 10.1182/blood-2017-02-767467 28546143

[22] Qiu Y, Zhang C, Shen K, et al. Clinical presentation and prognosis of light-chain amyloidosis patients with unmeasurable free light-chain levels. Ann Hematol. 2018 Dec;97(12):2465-2470. doi: 10.1007/s00277-018-3460-0 30056579

[23] Sidana S, Tandon N, Dispenzieri A, et al. Clinical presentation and outcomes in light chain amyloidosis patients with non-evaluable serum free light chains. Leukemia. 2018 Mar;32(3):729-735. doi: 10.1038/leu.2017.286 28919633

[24] Kastritis E, Papassotiriou I, Terpos E, et al. Clinical and prognostic significance of serum levels of von Willebrand factor and ADAMTS-13 antigens in AL amyloidosis. Blood. 2016 Jul 21;128(3):405-9. doi: 10.1182/blood-2016-02-702696 27166361

[25] Pudusseri A, Sanchorawala V, Sloan JM, et al. Prevalence and prognostic value of D-dimer elevation in patients with AL amyloidosis. Am J Hematol. 2019 Oct;94(10):1098-1103. doi: 10.1002/ajh.25576 31292986

[26] Kumar S, Dispenzieri A, Lacy MQ, et al. Serum uric acid: novel prognostic factor in primary systemic amyloidosis. Mayo Clin Proc. 2008 Mar;83(3):297-303. doi: 10.4065/83.3.297 18315995

[27] Yogo T, Okazuka K, Nashimoto J, et al. Red blood cell distribution width is a simple and novel biomarker for survival in light-chain amyloidosis. Int J Hematol. 2019 Oct;110(4):431-437. doi: 10.1007/s12185-019-02692-0 31236823

[28] Kastritis E, Papassotiriou I, Merlini G, et al. Growth differentiation factor-15 is a new biomarker for survival and renal outcomes in light chain amyloidosis. Blood. 2018 Apr 5;131(14):1568-1575. doi: 10.1182/blood-2017-12-819904 29386197

[29] Perfetti V, Colli Vignarelli M, Anesi E, et al. The degrees of plasma cell clonality and marrow infiltration adversely influence the prognosis of AL amyloidosis patients. Haematologica. 1999 Mar;84(3):218-21. 10189385

[30] Dinner S, Witteles W, Witteles R, et al. The prognostic value of diagnosing concurrent multiple myeloma in immunoglobulin light chain amyloidosis. Br J Haematol. 2013 May;161(3):367-72. doi: 10.1111/bjh.12269 23432783

[31] Tovar N, Rodríguez-Lobato LG, Cibeira MT, et al. Bone marrow plasma cell infiltration in light chain amyloidosis: impact on organ involvement and outcome. Amyloid. 2018 Jun;25(2):79-85. doi: 10.1080/13506129.2018.1443439 29482381

[32] Ozga M, Zhao Q, Benson D Jr, et al. AL amyloidosis: the effect of fluorescent in situ hybridization abnormalities on organ involvement and survival. Cancer Med. 2021 Feb; 10(3):965-73. doi: 10.1002/cam4.3683 33347707 PMC7897960

[33] Kobayashi H, Abe Y, Miura D, et al. Prevalence and clinical implications of t(11;14) in patients with amyloid light-chain amyloidosis with or without concurrent multiple myeloma. Jpn J Clin Oncol. 2019 Feb 1;49(2):195-198. doi: 10.1093/jjco/hyy202 30608548

[34] Bochtler T, Hegenbart U, Kunz C, et al. Translocation t(11;14) is associated with adverse outcome in patients with newly diagnosed AL amyloidosis when treated with bortezomib-based regimens. J Clin Oncol. 2015 Apr 20;33(12):1371-8. doi: 10.1200/JCO.2014.57.4947 25779559

[35] Bochtler T, Hegenbart U, Kunz C, et al. Prognostic impact of cytogenetic aberrations in AL amyloidosis patients after high-dose melphalan: a long-term follow-up study. Blood. 2016 Jul 28;128(4):594-602. doi: 10.1182/blood-2015-10-676361 27257181

[36] Sidana S, Tandon N, Brady PA, et al. Prognostic significance of Holter monitor findings in patients with light chain amyloidosis. Mayo Clin Proc. 2019 Mar;94(3):455-464. doi: 10.1016/j.mayocp.2018.08.039 30718070

[37] Nagano N, Yano T, Fujita Y, et al. Assessment of prognosis in immunoglobulin light chain amyloidosis patients with severe heart failure: a predictive value of right ventricular function. Heart Vessels. 2020 Apr;35(4):521-530. doi: 10.1007/s00380-019-01513-y 31559459

[38] Buss SJ, Emami M, Mereles, D, et al. Longitudinal left ventricular function for prediction of survival in systemic light-chain amyloidosis: incremental value compared with clinical and biochemical markers. J Am Coll Cardiol. 2012 Sep 18;60(12):1067-76. doi: 10.1016/j.jacc.2012.04.043 22883634

[39] Pun SC, Landau HJ, Riedel ER, et al. Prognostic and added value of two-dimensional global longitudinal strain for prediction of survival in patients with light chain amyloidosis undergoing autologous hematopoietic cell transplantation. J Am Soc Echocardiogr. 2018 Jan;31(1):64-70. doi: 10.1016/j.echo.2017.08.017 29111123 PMC5985664

[40] Lei C, Zhu X, Hsi DH, et al. Predictors of cardiac involvement and survival in patients with primary systemic light-chain amyloidosis: roles of the clinical, chemical, and 3-D speckle tracking echocardiography parameters. BMC Cardiovasc Disord. 2021 Jan 21;21(1):43. doi: 10.1186/s12872-021-01856-3 33478398 PMC7819214

[41] Mohty D, Boulogne C, Magne J, et al. Prognostic value of left atrial function in systemic light-chain amyloidosis: a cardiac magnetic resonance study. Eur Heart J Cardiovasc Imaging. 2016 Sep;17(9):961-9. doi: 10.1093/ehjci/jew100 27194782

[42] Arenja N, Andre F, Riffel JH, et al. Prognostic value of novel imaging parameters derived from standard cardiovascular magnetic resonance in high risk patients with systemic light chain amyloidosis. J Cardiovasc Magn Reson. 2019 Aug 22;21(1):53. doi: 10.1186/s12968-019-0564-1 31434577 PMC6704553

[43] Wan K, Lin J, Guo X, et al. Prognostic value of right ventricular dysfunction in patients with AL amyloidosis: comparison of different techniques by cardiac magnetic resonance. J Magn Reson Imaging. 2020 Nov;52(5):1441-1448. doi: 10.1002/jmri.27200 32691470

[44] Tahir UA, Doros G, Kim JS, Connors LH, Seldin DC, Sam F. Predictors of Mortality in Light Chain Cardiac Amyloidosis with Heart Failure. Sci Rep. 2019 Jun 12;9(1):8552. doi: 10.1038/s41598-019-44912-x 31189919 PMC6561903

[45] Mohty D, Pradel S, Magne J, et al. Prevalence and prognostic impact of left-sided valve thickening in systemic light-chain amyloidosis. Clin Res Cardiol. 2017 May;106(5):331-340. doi: 10.1007/s00392-016-1058-x 27933393

[46] Cho H, Kim S-J, Shim CY, et al. Prognostic significance of interventricular septal thickness in patients with AL amyloidosis. Leuk Res. 2017 Sep;60:36-43. doi: 10.1016/j.leukres.2017.06.008 28648672

[47] Kim D, Lee GY, Choi J-O, Kim K, Kim SJ, Jeon E-S. Associations of electrocardiographic parameters with left ventricular longitudinal strain and prognosis in cardiac light chain amyloidosis. Sci Rep. 2019 May 23;9(1):7746. doi: 10.1038/s41598-019-44245-9 31123293 PMC6533364

[48] Medvedovsky AT, Pollak A, Shuvy M, Gotsman I. Prognostic significance of the frontal QRS-T angle in patients with AL cardiac amyloidosis. J Electrocardiol. Mar-Apr 2020;59:122-125. doi: 10.1016/j.jelectrocard.2020.02.001 32062381

[49] Li H, Wang Y, Lan P, et al. Electrocardiographic parameters and prognosis of renal light chain amyloidosis. Clin Cardiol. 2020 Oct;43(10):1160-1166. doi: 10.1002/clc.23426 33460229 PMC7534015

[50] Stamatelopoulos K, Georgiopoulos G, Athanasouli F, et al. Reactive vasodilation predicts mortality in primary systemic light-chain amyloidosis. Circ Res. 2019 Sep 27;125(8):744-758. doi: 10.1161/CIRCRESAHA.119.314862 31401949 PMC6784773

[51] Binder C, Duca F, Binder T, et al. Prognostic implications of pericardial and pleural effusion in patients with cardiac amyloidosis. Clin Res Cardiol. 2021 Apr;110(4):532-543. doi: 10.1007/s00392-020-01698-7 32914241 PMC8055634

[52] Shen K-N, Miao H-L, Zhang C-L, et al. Posttreatment dFLC less than 10 mg/L predicts superior organ response and longer time to next treatment in newly diagnosed light-chain amyloidosis patients treated with bortezomib. Leuk Lymphoma. 2021 Apr;62(4):874-882. doi: 10.1080/10428194.2020.1849675 33215569

[53] Vaxman I, Sidiqi MH, Al Saleh AS, et al. Depth of response prior to autologous stem cell transplantation predicts survival in light chain amyloidosis. Bone Marrow Transplant. 2021 Apr;56(4):928-935. doi: 10.1038/s41409-020-01136-2 33208916

[54] Muchtar E, Dispenzieri A, Jevremovic D, et al. Survival impact of achieving minimal residual negativity by multi-parametric flow cytometry in AL amyloidosis. Amyloid. 2020 Mar;27(1):13-16. doi: 10.1080/13506129.2019.1666709 31544536 PMC7372715

